# Clinical evaluation the success rate and complications of fluoroscopically guided removal of tracheal tube metallic stents

**DOI:** 10.1186/s13019-021-01444-8

**Published:** 2021-03-25

**Authors:** Zong-Ming Li, De-Chao Jiao, Xin-Wei Han, Hui-Bin Lu, Ke-Wei Ren, Hong Yang

**Affiliations:** Department of Interventional Radiology, the First Affiliated Hospital of Zhengzhou University, Zhengzhou, 450052 China

**Keywords:** Tracheal, Tubular, Stent, Removal

## Abstract

**Background:**

Long-term placement of airway stents has a high probability of restenosis of the airway due to granulation tissue hyperplasia, and it is difficult to remove the stent. Our aim is to evaluate the success rate and complications of removal of tracheal tube metallic stents under fluoroscopic guidance, and to compare the difference between uncovered stent and covered stent.

**Methods:**

We retrospectively reviewed 45 cases (31 males and 14 females; age, 12–71 years) of tracheal metallic stent removal performed at our center between January 2014 and December 2019. Covered stents were applied in 36 cases, and uncovered stents were applied in 9 cases. In the covered stent group, 15 patients presented with granulation tissue at both ends; 3 cases, with stent fracture; and 2, with stent intolerance due to severe airway foreign body sensation. In the uncovered stents group, all patients presented with granulation tissue formation; 2 patients, with stent fracture; and 1 patient, with stent intolerance.

**Results:**

A total of 41 (91.1%) stents were successfully removed (34 [94.4%] in the covered stent group and 7 [77.8%] in the uncovered stent group). The average duration of stent placement was 3.2 ± 0.7 and 2.5 ± 1.2 months in the covered stent group and uncovered stent group, respectively. With regard to the complications, hemoptysis occurred in 4 cases (average blood volume lost, 100 ml), tracheal mucosa tear occurred in 5 cases, tracheal collapse requiring emergency airway stent placement occurred in 1 case, and tracheal rupture requiring emergency surgical suture occurred in 1 case. No procedure-related deaths occurred in either group.

**Conclusions:**

It is safe to remove the metal stent of the tracheal tube under the guidance of fluoroscopy, with low complications, and can avoid the long-term placement of the airway stent.

## Background

Airway stenting treatment of benign and malignant airway stenosis is an effective approach that has been used widely in clinical practice [1]. The popularity of airway stent treatment has been growing among clinicians because it is minimally invasive and immediately effective [2]. However, foreign body stimulation after stent placement can impact expectoration function and, subsequently, result in sputum retention, abrasion of the airway membrane that causes bleeding, formation of granulation tissue that results in airway restenosis, and other complications. Especially in patients with benign airway disease, who have longer survival, long-term airway stent implantation is associated with several complications [3]. Some of the reported signs of these complications are severe cough, sputum retention, bleeding, and airway restenosis caused by granulation tissue obstructing the stent. Therefore, temporarily placed airway stents need to be removed after successful clinical treatment of airway stenosis, especially in patients with benign airway lesions. However, there are few literatures about airway stent removal, and most of them are case reports. Mostly under bronchoscopy, the upper end of the stent is clamped and rotated with biopsy forceps, and then the stent is removed [4, 5]. This method is easy to cause the stent to break and mucosal damage and bleeding.

## Methods

### Clinical data

Between January 2014 and December 2019, removal of tracheal tube metallic stents was performed in 45 patients at the Interventional Treatment Center of our hospital. The cohort, which included 31 males and 14 females (12–71 years) had undergone tracheal stent insertion (with 36 covered stents and 9 uncovered stents) for various tracheal diseases. In the covered stent group, the reasons for stent removal were the formation of granulation tissue at both ends of the stent in 15 cases, stent fracture in 3 cases, and inability to tolerate the stent in 2 cases due to severe airway foreign body sensation. In the uncovered stent group, the reasons were granulation tissue in all cases, stent fracture in 2 cases, and inability to tolerate the stent in 1 case. The mean duration of stent placement in the covered group and uncovered group was 3.2 ± 0.7 and 2.5 ± 1.2 months respectively. Detailed information about all the patients is presented in Table 1.

**Table 1:** Case characteristics

Disease	Covered group (*n* = 36)	Uncovered group (*n* = 9)
Cicatricial tracheal stenosis after tracheotomy	12	3
Granulation tissue hyperplasia after tracheotomy	3	1
Cicatricial stenosis of the trachea after tracheal intubation	9	2
Granulation tissue hyperplasia after tracheal intubation	1	0
Tracheobronchial tuberculosis	4	2
Traumatic injuries	1	0
Polychondritis	1	0
Tracheal invasion by esophageal carcinoma	2	1
Esophageal stent oppression	1	0
Lung cancer	1	0
Neoplasm metastasis or lymphadenectasis	1	0

Written informed consent was obtained from the patient for the publication of this article and any accompanying images. We also obtained the permission of the Ethics Committee of our center.

### Preoperative evaluation

**Fig. 1:**
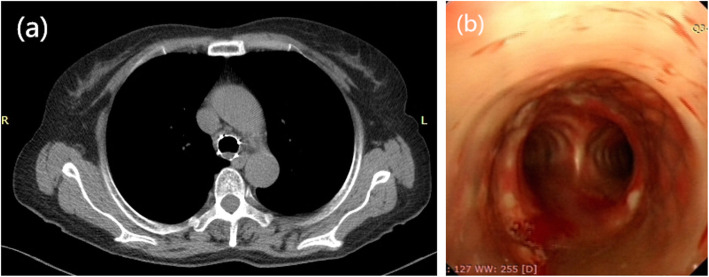
Chest CT (**a**) and bronchoscopy (**b**) before tracheal stent removal

The preoperative evaluations included routine blood tests, liver and kidney function tests, serum electrolyte tests, communicable disease tests, and blood coagulation tests. The imaging examinations included thoracic computed tomography (CT) and bronchoscopy (Fig. 1a & b), as well as electrocardiography. Stents with granulation tissue at both ends and stents that were completely embedded in the submucosal layer were exposed with the help of high-frequency electric cautery or argon cryotherapy under bronchoscopic guidance.

### Equipment and drugs

The equipment included a 5F angiography catheter (COOK, USA), a 0.035-in. hydrophilic membrane guidewire (Terumo Corp, Japan), a 0.035-in. strengthening guidewire (COOK, USA), a 12F sheath (COOK, USA), a stent removal hook (Nanjing MicroPort, China), opener, and an ECG suction device.

The patients were administered lidocaine (as an anesthesia-inducing agent) and epinephrine (as a homeostasis-restoring agent).

7# intubations and ventilators were employed as alternating options.

### Tracheal stent removal

Fluoroscopy table was performed with the patients in the supine position. After the necessary preparations for oxygen inhalation, ECG monitoring, spraying of the throat with lidocaine, and vacuum suctioning to clear the airway and oral secretions, the shoulder and neck of the patient were elevated so that they were facing right.

Under fluoroscopic guidance, an angiographic catheter was passed over a guide wire through the mouth, pharynx, larynx, and trachea, and then into the right main bronchus, and the guide wire was removed. A 2–3 ml bolus of 2% lidocaine was injected through the catheter, following which 2–3 ml of 0.02% epinephrine was administered to prevent bleeding.

**Fig. 2:**
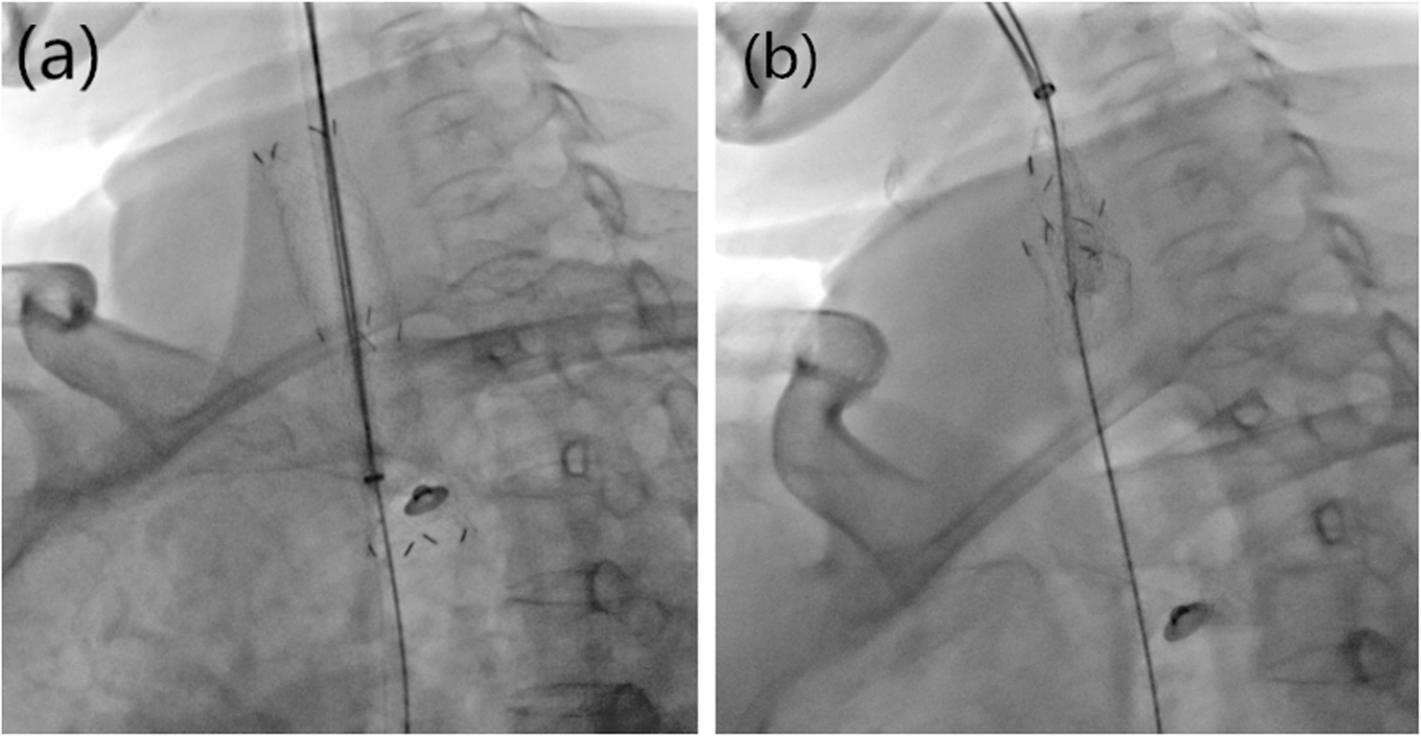
Position of the stent retrieval hook in relation with the sheath for hooking the metal wire to the lower end of the tracheal stent, while maintaining the relative position of the hook and sheath (**a**), external pulling of the stent retrieval hook and sheath leading to inward folding and exfoliation of the stent, and finally, retrieval of the stent (**b**)

A strengthening guidewire was introduced into one main bronchus. A 12F sheath was then sent out along the strengthening guidewire after the catheter was dropped out and the inner core was pulled out. A stent retrieval hook was then introduced through the sheath with extension of the sheath tip by 2 cm. Next, the direction of the stent retrieval hook was adjusted so that it hooked the metal wire at the lower end of the tracheal stent (Fig. 2a). The relative position of the stent retrieval hook and sheath were maintained, and the strengthening guidewire was fixed. Next, the stent retrieval hook was pulled out such that the stent folded inwards and was exfoliated (Fig. 2b), and it was then pulled out. Finally, a catheter was introduced along the guidewire, and 2–3 ml of 0.02% epinephrine was sprayed through the catheter for maintenance of hemostasis.

The vital signs of the patients were closely observed intraoperatively. If suffocation or massive hemoptysis occurred, emergency procedures such as endotracheal intubation and tracheostomy, or mechanical ventilation, were performed as necessary.

### Complications management

For patients with hemoptysis, first push in epinephrine diluted saline (0.1 mg/ml) through the catheter to stop bleeding. If the hemostatic effect is not satisfactory, then perform bronchial angiography and embolization therapy. For patients with severe dyspnea caused by tracheal collapse, the airway stent should be re-inserted immediately to relieve symptoms. For tracheal ruptures with a low incidence, surgical thoracotomy and repair of the ruptured trachea should be accepted as well.

### Treatment and follow-up after stent removal

**Fig. 3:**
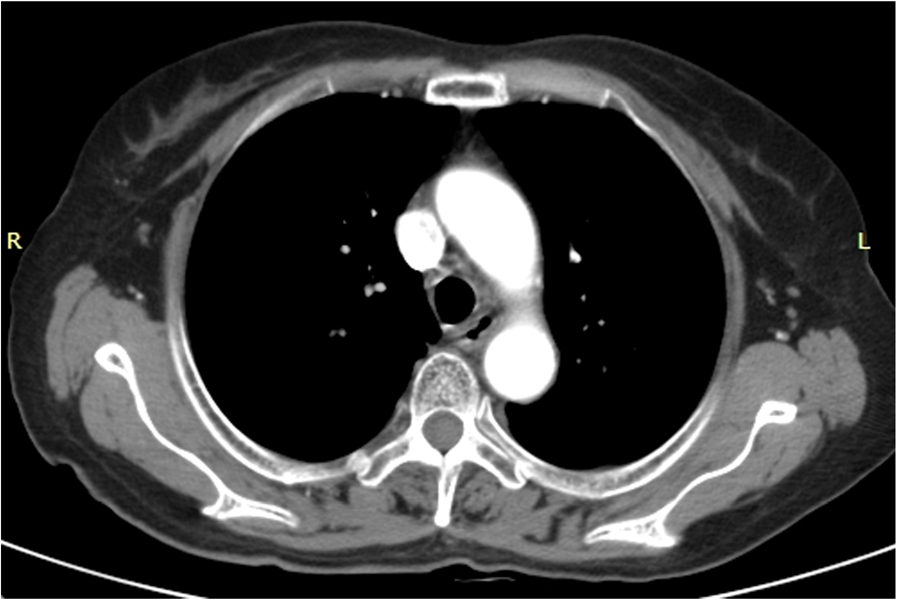
Chest CT scan showing tracheal patency after tracheal stent removal

All patients received aerosol inhalation 2 to 3 times a day to reduce inflammation after the stent removed; additionally, anti-inflammatory, anti-phlegm, and other symptomatic treatments were administered as required. Chest CT was performed 3 days after stent removal to observe the recovery of tracheal stenosis or fistula formation (Fig. 3). Bronchoscopy was performed 3 days, 7 days, 1 month, and 3 months after stent removal to observe tracheal mucosal damage and the clearing of necrotic tissue and granulation tissue under bronchoscopic guidance, as necessary.

### Statistical analysis

Data were processed and analyzed by GraphPad Prism 5.0. The data obtained were compared with the *t*-test. Count data were compared with the χ^2^ test. *P* values < 0.05 were considered to indicate statistical significance.

## Results

In the covered stent group, 15 cases presented with granulation tissue at both ends; 3 cases, with stent fracture; and 2 cases, with stent intolerance. In the uncovered stent group, all cases presented with granulation tissue; 2 cases, with stent fracture; and 1 case, with stent intolerance. Out of the 45 stents, 41 (91.1%) stents were successfully removed: 34 (94.4%) stents were successfully removed in the covered stent group, and 7 (77.8%) stents were successfully removed in the uncovered stent group (Table 2).

**Table 2:** Details of the stent removal procedure in the covered stent group and uncovered stent group

Stent type	Number of stents	Preoperative features		Postoperative features				Without removal
		Granulation tissue	Stent fracture	Com-plete	Damage	Debris	Total	
Covered stent group	36	15	3	27	5	2	4	2
Uncovered stent group	9	9	2	2	3	2	7	2

In the covered stent group, hemoptysis occurred in 2 cases (average hemorrhage volume, 80 ml) and tracheal mucosal tear occurred in 2 cases, but there were no procedure-related deaths. In the uncovered stent group, hemoptysis occurred in 2 cases (average hemorrhage volume, 120 ml), tracheal mucosa tear occurred in 3 cases, tracheal collapse requiring immediate airway stent placement occurred in 1 case, and tracheal rupture requiring emergency surgical suture occurred in 1 case. There were no procedure-related deaths in this group either. In each of the groups, the tracheal stent could not be removed in 2 cases. One patient refused to undergo stent removal, and in another case, the stent was not removed due to cannot tolerate airway stent removal. In the remaining two cases, the stents were not removed because of severe obstruction by granulation tissue.

The degree of granulation tissue formation was significantly lower in the covered stent group than in the uncovered stent group (*P* = 0.0018), but the incidence of stent fracture was not significantly different between the two groups (*P* = 0.2575). There was a significant difference between the two groups in terms of the integrity of the stent after removal (*P* = 0.0071). There was no significant difference between the two groups with regard to stent fracture and debris formation (*P* = 0.0870 and 0.0654, respectively).

During follow-up in the covered stent group, restenosis occurred in 5 cases at 1 month after stent removal. In 4 cases, significant improvement was observed after high-frequency electrical excision and cryotherapy with argon. In another case of recurring granulation tissue, improvement was observed after permanent tracheal stent placement. In one case, mild dyspnea occurred due to tumor recurrence at 1 month after stent removal, and it improved after high-frequency electrical excision under bronchoscopic guidance combined with systemic chemotherapy. In the uncovered stent group, granulation tissue formation occurred in 2 cases and significantly improved after high-frequency electrical excision and cryotherapy with argon under bronchoscopic guidance.

## Discussion

In the present study, we describe the removal of tracheal metallic stents under fluoroscopic guidance with a high success rate (91.1%). These findings are important, as there are few reports on tracheal stent removal after stent placement.

The shorter the duration of airway stent placement, the lower is the degree of proliferation of granulation tissue, and the easier it is to remove the stent. However, airway plastic remodeling is inadequate, and the rate of airway restenosis is greater after stent removal. On the other hand, the longer the duration of airway stent placement, the more adequate is the rate of plastic remodeling of the airway, and the lower is the rate of airway restenosis after stent removal. However, when the rate of proliferation of granulation tissue is higher, stent removal is easier and the rate of complications is higher. Studies [5, 6] have shown that the time required for plastic remodeling of scar tissue is about 3 months. This matches the mean duration of covered stent and uncovered stent placement (which was 3.2 ± 0.7 and 2.5 ± 1.2 months respectively) in the present study.

Compared with the uncovered stent group, in the covered stent group, the rates of granulation tissue and stent fracture were lower and the success rate of stent removal was higher. Therefore, we recommend the use of covered tracheal stents in patients who need a temporary tracheal stent, especially those with benign disease.

In the present study, chest CT was performed before stent removal to determine the position of the tracheal stent, and bronchoscopy was used to examine the formation of granulation tissue at both ends of the stent. When there was severe granulation tissue formation, high-frequency electric cutting and argon-laser cryotherapy under bronchoscopic guidance were used to clear excessive granulation tissue. Additionally, the remaining metal wire of the stent can be used for stent removal. In preparation for stent removal, anti-inflammatory therapy, including inhalation of anti-inflammatory drugs to reduce airway inflammation and promote mucus discharge, was administered.

Highly skilled surgeons and adequate hemostasis are critical factors that affect the successful retrieval of tracheal stents. The risks associated with airway stent removal are greater than those associated with stent placement, as the former procedure requires that the surgeon master the technical aspects of both stent placement and removal [7]. When part of the stent or wire is embedded in the granulation tissue, a stent retrieval hook is hooked onto the lower end of stent, and the stent is then pulled so that it folds and is stripped out of the endotracheal tube [8]. In such patients, lesser resistance and tracheal wall damage make it easier to remove the stent. When the stent retrieval hook is hooked onto the metal wire, doctors should pay attention to the length of hook protruding into the sheath in order to avoid airway wall perforation, which may cause mediastinal abscess formation. In the present study, hemostasis maintenance drugs were sprayed via the catheter at the location of stent placement before and after stent removal to prevent tissue bleeding.

The present study is limited by its small sample size, its retrospective design, and lack of long-term follow-up and animal experiments. Additionally, we have limited experience with this procedure. In particular, further studies are needed to determine the optimal time of stent removal.

## Conclusion

Airway restenosis caused by granulation tissue hyperplasia is the most common complication after airway stent placement. Therefore, after the airway reconstruction and shaping, remove the stent to avoid long-term indwelling of the airway stent, which greatly reduces the occurrence of long-term complications. For benign airway stenosis, a covered stent is recommended. The removal of the airway stent with the sheath and the stent removal hook under fluoroscopy has little trauma and low complication rate, which is worth promoting.

## Data Availability

The datasets generated and analyzed during the current study are available from the corresponding author on reasonable request.

## Abbreviations

CT: Computed Tomography
ECG: Electrocardiogram
